# A new species of parasitic copepod, *Sarcotretes umitakae* sp. n. (Siphonostomatoida, Pennellidae), on the rattail (Actinopterygii, Macrouridae) from the East China Sea, Japan

**DOI:** 10.3897/zookeys.246.3872

**Published:** 2012-11-29

**Authors:** Daisuke Uyeno, Kaori Wakabayashi, Kazuya Nagasawa

**Affiliations:** 1 Faculty of Science, University of the Ryukyus, 1 Senbaru, Nishihara, Okinawa 903-0213, Japan; 2 Faculty of Marine Science, Tokyo University of Marine Science and Technology, 4–5–7 Konan, Minato, Tokyo 108–8477, Japan; 3 Graduate School of Biosphere Science, Hiroshima University, 1–4–4 Kagamiyama, Higashi–Hiroshima, Hiroshima 739–8528, Japan

**Keywords:** Mesoparasitic copepods, *Sarcotretes umitakae* sp. n., new species, East China Sea, rattail, mesopelagic fishes

## Abstract

A new species of copepod, *Sarcotretes umitakae* **sp. n.**, of the siphonostomatoid family Pennellidae is described based on female specimens from the rattail *Coelorinchus jordani* Smith and Pope (Actinopterygii: Gadiformes: Macrouridae) caught in the East China Sea. This species is characterized by exhibiting the following characters: the large proboscis projects strongly; the head bears paired lateral processes which are bulbous and taper into a slender horn; the twisting neck is significantly longer than the trunk; and the trunk bears an anterior constriction with a reduced abdomen.

## Introduction

*Sarcotretes* Jungersen, 1911, a pennellid genus, was originally established based on *Sarcotretes scopeli* Jungersen, 1911 from Ireland, the eastern North Atlantic (Jungersen 1911). Wilson (1917) included six species in this genus: *Sarcotretes scopeli* (type species), *Sarcotretes eristaliformis* (Brian, 1908), *Sarcotretes gempyli* (Horst, 1879), *Sarcotretes inflexus* (Steenstrup and Lütken, 1861), *Sarcotretes nodicornis* (Steenstrup and Lütken, 1861), and *Sarcotretes lobatus* Wilson C.B., 1917. Subsequently, *Sarcotretes inflexus*, *Sarcotretes nodicornis*, *Sarcotretes gempyli*, and *Sarcotretes lobatus* were considered to be junior synonyms of *Sarcotretes scopeli* by Hogans (1988). Recently, *Sarcotretes longirostris* Ho, Nagasawa and Kim, 2007 was described, thus a total of three species are considered as valid in this genus at present (Ho et al. 2007). Members of the genus are parasitic on mesopelagic fishes (Cherel and Boxshall 2004), and they were often been found in food samples of penguins and whales (e.g. Cherel and Boxshall 2004; Ho et al. 2007). In this study, a new species of the genus is described based on females from the rattail *Coelorinchus jordani* Smith and Pope (Actinopterygii: Gadiformes: Macrouridae) caught in the mesopelagic zone of the East China Sea.

## Materials and methods

Two specimens of the rattail *Coelorinchus jordani* infected with copepods were caught in the East China Sea off the Tokara Islands, Kagoshima, Japan on 8 October 2011 during the cruise (UM-11-06) of the *Umitaka-maru*, a training and research vessel of Tokyo University of Marine Science and Technology (TUMSAT). The fishes were collected using an otter trawl towed for 30 minutes between two sites (29°58.02'N, 127°43.79'E to 29°59.57'N, 127°44.28'E) around 309 m in depth and, immediately after capture, they were preserved in 70% ethanol with copepods attached. In the laboratory, copepods were carefully removed from the tissues of fishes, and then soaked in lactophenol for a whole day before dissection. The appendages of the copepods were observed after dissecting with the method of Humes and Gooding (1964). Drawings were made with the aid of a drawing tube. Morphological terminology follows Huys and Boxshall (1991). Measurements (in millimeters) are shown as ranges, followed by means and standard deviations in parentheses. The flexed part was measured along the body axis. Type specimens are deposited in the crustacean collection at the National Museum of Nature and Science, Tsukuba (NSMT). The scientific name of fish follows Nakabo (2002).

## Results

### Order Siphonostomatoida Burmeister, 1835

Family Pennellidae Burmeister, 1835

Genus *Sarcotretes* Jungersen, 1911

#### Sarcotretes umitakae sp. n.

urn:lsid:zoobank.org:act:879FD1B6-AC17-4DD0-9E27-AFD4EF2A4DEF

http://species-id.net/wiki/Sarcotretes_umitakae

**Figure 1.**
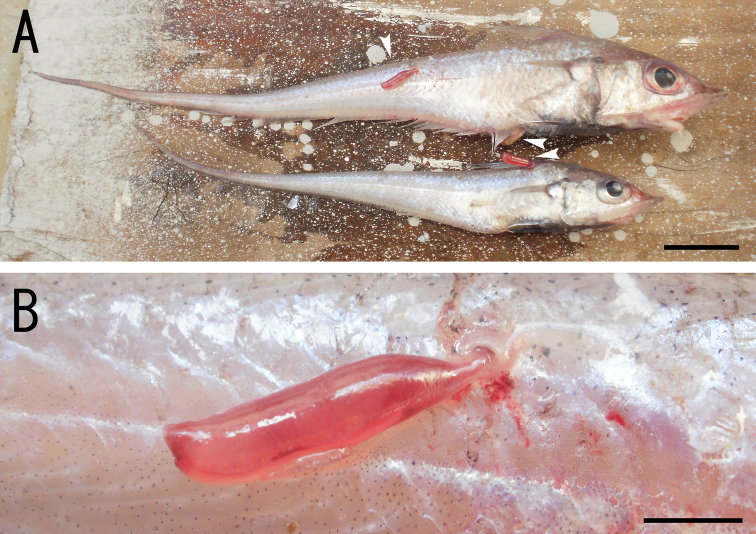
*Sarcotretes umitakae* sp. n., female on *Coelorinchus jordani* Smith and Pope. **A** two specimens of *Coelorinchus jordani* (181.5 mm TL and 142.8 mm TL) carrying the type series of *Sarcotretes* sp. n. (arrowheads) **B** coloration in life of paratype NSMT–Cr 22254 attached to host’s body. Scale bars: **A**=20 mm; **B**=3 mm.

Figures 1 [Fig F2] [Fig F3] 4

##### Type material.

Holotype female (NSMT–Cr 22253) and 2 paratypic females (NSMT–Cr 22254), ex *Coelorinchus jordani* Smith and Pope (Gadiformes: Macrouridae), taken off the Tokara Islands (29°58.02'N, 127°43.79'E to 29°59.57'N, 127°44.28'E), Kagoshima, East China Sea, Japan, 308.5–309.3 m depth, 8 October 2011, reg. K. Wakabayashi and Y. Tanaka.

##### Type locality.

off the Tokara Islands (29°58.02'N, 127°43.79'E to 29°59.57'N, 127°44.28'E), Kagoshima, East China Sea, Japan.

##### Description of postmetamorphic adult female.

**Figure 2. F2:**
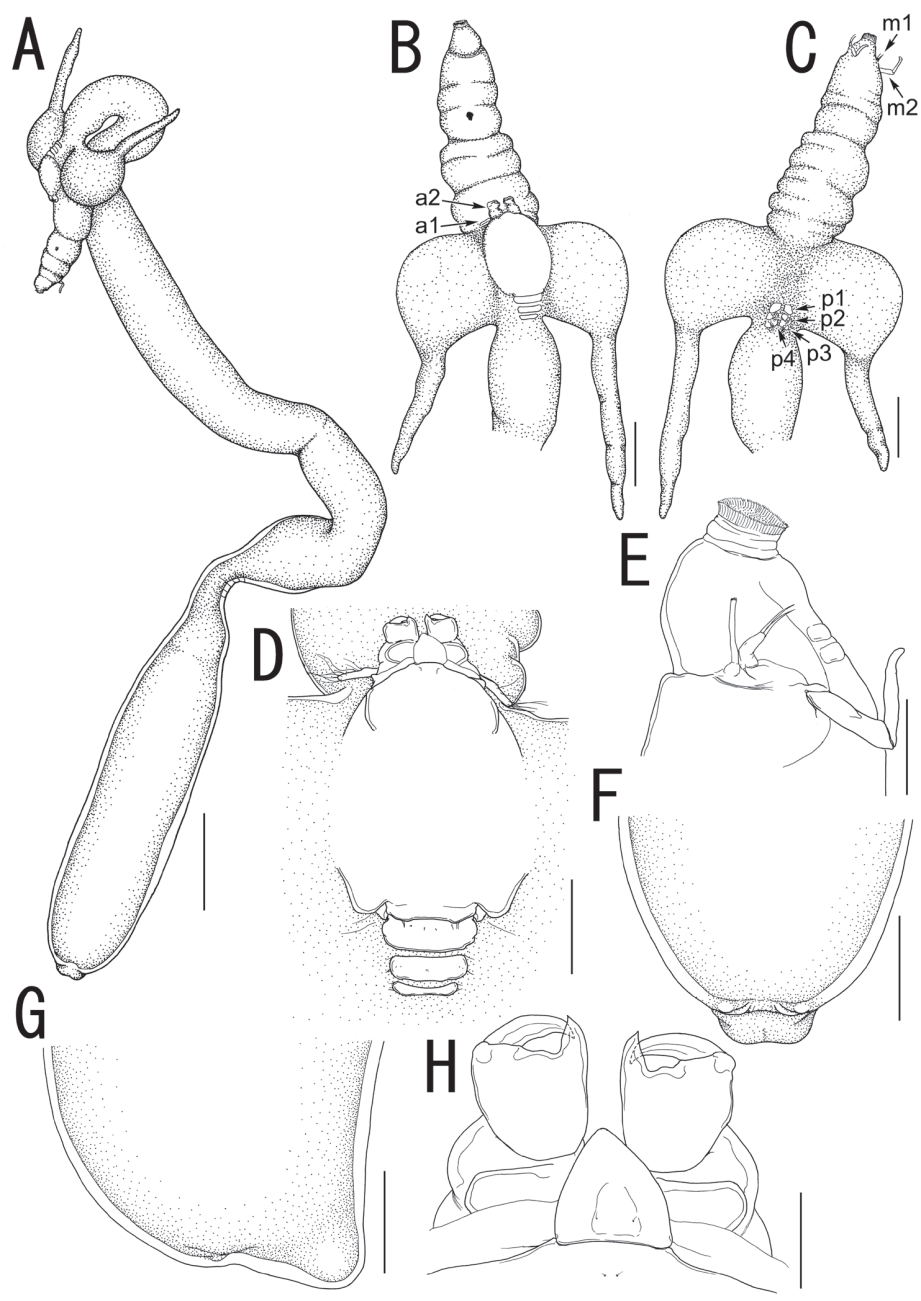
*Sarcotretes umitakae* sp. n., female, holotype NSMT–Cr 22253. **A** habitus **B** anterior portion of body, dorsal, a1 = antennule, a2 = antenna **C** same, ventral, m1 = maxillule, m2 = maxilla, p1 = leg 1, p2 = leg 2, p3 = leg 3, p4 = vestige of leg 4 **D** vestige of dorsal cephalothoracic shield **E** tip of proboscis, lateral **F** posterior portion of body, ventral **G** same, lateral **H** rostral area and antennae, dorsal. Scale bars: **A**=3 mm; **B, C, F, G**=1 mm; **D**=500 μm; **E**=300 μm; **H**=150 μm.

Body (Fig. 2A) elongate, comprising head, neck, and trunk. Total length 43.42 (from tip of cephalothorax to end of abdomen). Head (holdfast) composed of cephalosome to third pediger (Fig. 2A, B), bearing elongate oral area projecting forward as cylindrical proboscis with multiple constrictions and mouth tube at its tip, with paired lateral processes (Fig. 2A, B) consisting of bulbous base drawn out into highly sclerotized horn-like process. Vestige of dorsal shield of cephalothorax and tergites of first to third visible on dorsal surface of head (Fig. 2D). Two paired small sclerites on ventral surface of basal region of oral cone (Fig. 2E). Neck (Fig. 2A) slender, longer than trunk, twisting and bearing bulge and constriction at posterior portion. Cylindrical trunk (Fig. 2A) 12.23 long (from enlarged end of neck to abdomen), 3.08 wide at widest part bearing paired hemispherical protrusions and reduced abdomen (Fig. 2F, G). Caudal rami absent.

**Figure 3. F3:**
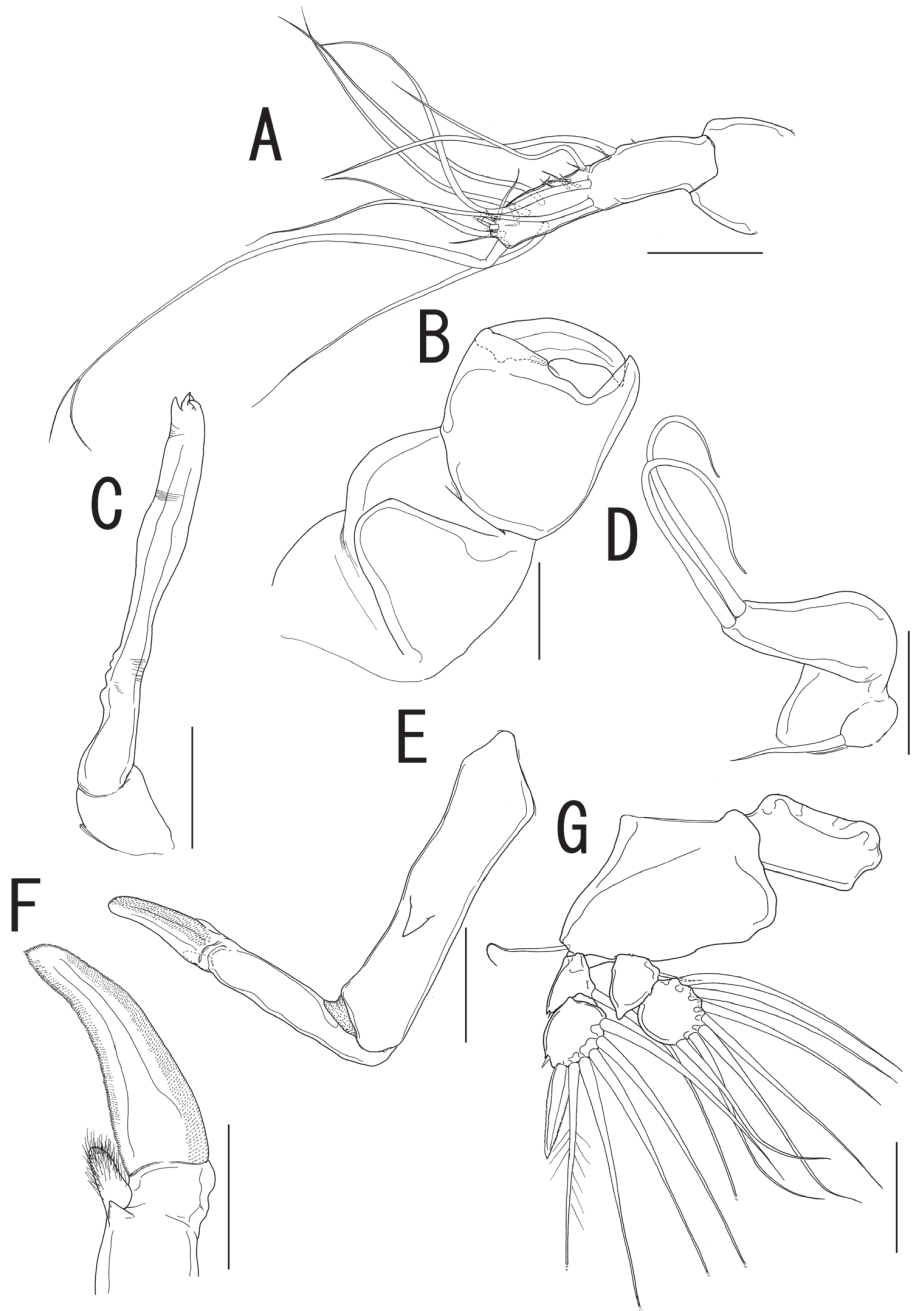
*Sarcotretes umitakae* sp. n., female, holotype NSMT–Cr 22253. **A** left antennule, anterior **B** left antenna, anterior **C** left mandible **D** left maxillule **E** left maxilla, lateral **F** distal part of left maxilla **G** right leg 1, anterior. Scale bars: **A, B, E, G**=100 μm; **C, D**=70 μm; **F**=50μm.

Rostral area (Fig. 2H) triangular. Antennule (Fig. 3A) not segmented, located on sclerotized protrusion, bearing 10 blunt, long elements, and at least 17 short elements; 1 long distal seta with bifurcated tip. Antenna (Fig. 3B) subchelate, 3-segmented; proximal segment, unarmed; middle segment stout with a pointed process on innerdistal corner, hollowed out to receive terminal claw; terminal segment representing terminal claw with single basal seta. Mandible (Fig. 3C) located on lateral side of base of oral cone (Fig. 2E), represented by sclerotized process with unequal processes tip. Maxillule (Fig. 3D) bilobate; large inner lobe tipped with two naked setae; small outer lobe bearing one naked seta. Maxilla (Fig. 3E) 2-segmented; proximal segment rod-like, bearing round protrusion with pointed process in middle portion; terminal segment rod-like, tipped with curved spinulose process, small pointed process, and setulous lobe (Fig. 3F). Maxilliped absent.

**Figure 4.**
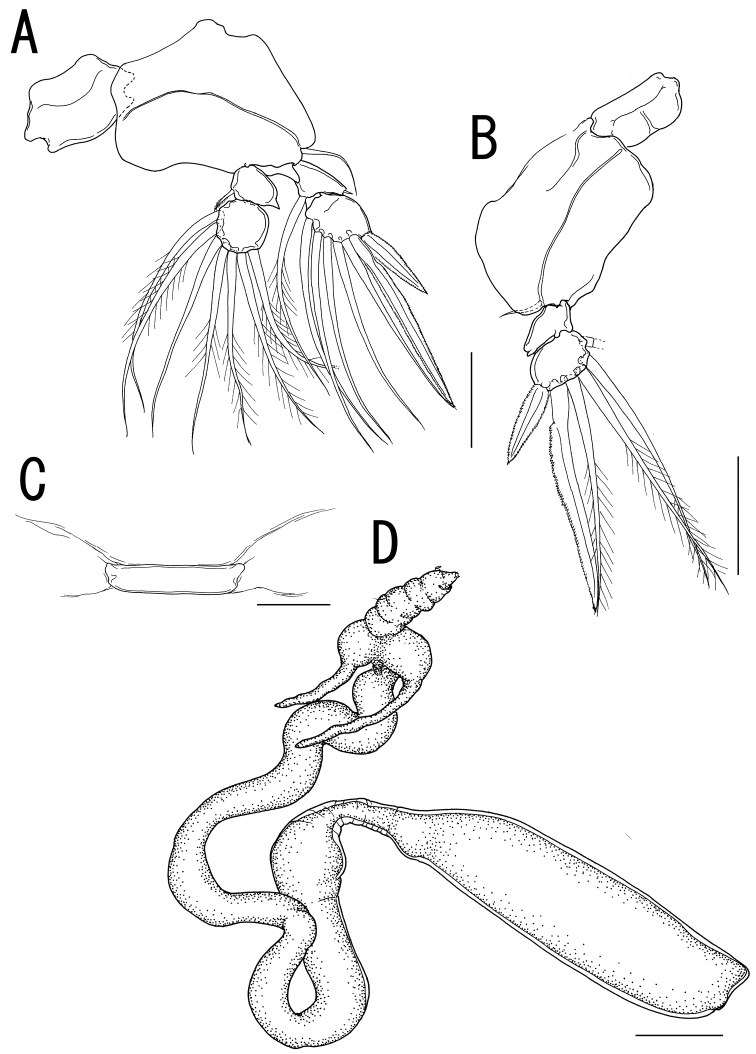
*Sarcotretes umitakae* sp. n., female, holotype NSMT–Cr 22253. **A** left leg 2, anterior **B** right leg 3, anterior **C** vestige of leg 4. *Sarcotretes umitakae* sp. n., female, paratype NSMT–Cr 22254 **D** habitus. Scale bars: **A, B**=100 μm; **C**=30 μm; **D**=3 mm.

Legs 1 to 4 occurring tightly together and located between paired lateral processes of holdfast. Legs 1 and 2 (Figs. 3G, 4A) biramous, composed of inter coxal sclerites, protopods, and 2-segmented rami. Leg 3 (Fig. 4B) uniramous, without endopod; leg armature formula as follows:

Leg 4 (Fig. 4C) represented by vestigial intercoxal sclerite. Legs 5 and 6 absent.

*Variability of female*. The necks of all paratypes twist and turn in complex fashion (Fig. 4D). Measurements of the body parts of the specimens from the type series (n = 3) are as follows: body length (anterior margin of the head to distal end of the posterior processes on the trunk) 30.26–50.12 (41.27 ± 10.10); trunk length 11.27–13.48 (12.33 ± 1.11); trunk width 2.84–3.17 (3.03 ± 0.17).

Male. Unknown.

##### Site.

The copepod attaches to various parts of the body surface of the host fishes (Fig. 1A). The head to the neck of the copepod was embedded in the host’s musculature, and the trunk was protruded into the water (Fig. 1B)

##### Etymology.

The specific name “*umitakae”* refers to the *Umitaka-maru*, a training and research vessel of TUMSAT.

##### Remarks.

Currently, three species of *Sacotretes*: *Sacotretes eristaliformis*, *Sacotretes longirostris*, and *Sarcotretes scopeli*, are considered to be valid (Ho et al. 2007). *Sarcotretes umitakae* sp. n. differs from *Sarcotretes eristaliformis* and *Sarcotretes scopoli* by having the holdfast with paired bulbous swellings drawn out into an elongate, horn-like process (vs. bulbous with or without a blunt tip) and the neck approximately 3 times as long as the trunk (vs. nearly as long as the trunk in *Sarcotretes eristaliformis* and less than 3/4 of the trunk length in *Sarcotretes scopeli*) (Brian 1912; Hogans 1988; Boxshall 1989; Cherel and Boxshall 2004; Ho et al. 2007). The two specimens described as *Sarcotretes eristaliformis* by Kabata and Gusev (1966) were judged to be *Sarcotretes scopeli* on the basis of a body length of 15.8–21.2 mm, the neck being shorter than the trunk, and relative length of the proboscis (Hogans 1988; Ho et al. 2007). In addition, *Sarcotretes scopeli* differs from the new species by the absence of the vestige of leg 4 (vs. presence) (Boxshall 1989; Cherel and Boxshall 2004). *Sarcotretes longirostris* has the neck longer than the trunk like *Sarcotretes umitakae* sp. n. and only these 2 species possess an extremely long proboscis among their congeners. *Sarcotretes longirostris* is, however, easily distinguishable from the new species by having the following characters: slender lateral head processes without swollen basal portions (vs. a bulbous swelling with a slender process); a not defined rostral area (vs. triangular); and a large and conical reduced abdomen, protruding to posterior (vs. small and non-conical and slightly protruded to posterodorsal) (Ho et al. 2007). In addition, the trunk has an anterior constriction in *Sarcotretes umitakae* sp. n. not shared with any of the three known species (Figs. 2A, 4D).

## Discussion

Despite the fact that some morphological characters of *Sarcotretes* species (e.g. the shape of the holdfast, the length and flexure of the neck, and the length of the proboscis) show variability, they have been conventionally used to distinguish the species in this genus (Hogans 1988). Because these characters vary according to the site of attachment to the host and the age of the parasite, species identification using such characters may not make sense. Actually, based on those characters, Wilson (1917) had recognized 6 species as valid, but later, 4 species (*Sarcotretes gempyli*, *Sarcotretes inflexus*, *Sarcotretes lobatus*, and *Sarcotretes nodicornis*) were regarded as junior synonyms of *Sarcotretes scopeli* by Hogans (1988). Nonetheless, we consider that such characters as the length of the proboscis and the shape of the lateral horns on the head, which were used in the key given by Wilson (1917), are useful identification characters. These characters do exhibit variability but there is no overlap between in *Sarcotretes umitakae* sp. n. and existing species. *Sarcotretes umitakae* sp. n. and *Sarcotretes longirostris* possess a strongly projecting proboscis which is not shared with other congeners. The lateral process of *Sarcotretes umitakae* sp. n. comprising a bulge with a pointed tip is similar to that of *Sarcotretes scopeli* and *Sarcotretes eristaliformis*, but the greatly elongated tip in *Sarcotretes umitakae* sp. n. is apparently distinguishable (see Brian 1912, pls. 9, 10; Hogans 1988, fig. 2b; Boxshall 1989, fig. 3; present study, Fig. 2). Although a great care is required, it is considered that these characters can provide reliable evidence of species identity. On the other hand, all three specimens of *Sarcotretes umitakae* sp. n. have a neck with a posterior protrusion and a constriction behind it. However, this character cannot be used to identify *Sarcotretes umitakae* sp. n. because a similar character was observed in some specimens of *Sarcotretes eristaliformis* and *Sarcotretes scopeli* (see Brian 1912; Boxshall 1989).

The discovery of *Sarcotretes umitakae* sp. n. in this study shows that there are at least 2 species of the genus in Japanese waters.

### Key to females of the species of *Sarcotretes*

**Table:** 

1	Proboscis slightly projecting; holdfast composed of broad base with or without terminal process; neck shorter than or as long as trunk	2
–	Proboscis elongate, strongly projecting; holdfast comprising slender processes; neck significantly longer than trunk	3
2	Body up to approximately 25 mm long; neck shorter than trunk; leg 4 absent	*Sarcotretes scopeli*
–	Body approximately 45 mm or longer (about twice length of *Sarcotretes scopeli*); neck about as long as trunk; vestige of leg 4 (intercoxal sclerite) present	*Sarcotretes eristaliformis*
3	Slender, horn-like lateral processes on head (holdfast); trunk not constricted; reduced abdomen conical, projecting posteriorly	*Sarcotretes longirostris*
–	Lateral process bulbous tapering into slender, elongate horn; trunk with anterior constriction; abdomen reduced, vestigial	*Sarcotretes umitakae* sp. n.

## Supplementary Material

XML Treatment for Sarcotretes umitakae
